# New Record of Pipefish from the Coast of Mainland China with Phylogeography and Conservation Insights

**DOI:** 10.3390/ani16081161

**Published:** 2026-04-10

**Authors:** Xin Wang, Hao Luo, Shuaishuai Liu, Zhixin Zhang, Qiang Lin

**Affiliations:** 1 State Key Laboratory of Tropical Oceanography, Guangdong Provincial Key Laboratory of Applied Marine Biology, South China Sea Institute of Oceanology, Chinese Academy of Sciences, Guangzhou 510301, China; 2 Sanya Marine Ecological Environment Engineering Research Institute, Sanya 572000, China; 3 University of the Chinese Academy of Sciences, Beijing 100049, China; 4 College of Life Science and Oceanography, Shenzhen University, Shenzhen 518060, China

**Keywords:** Syngnathidae, phylogeny, population genetic, genetic diversity, conservation

## Abstract

The evolutionary history and contemporary biogeography jointly shape the genetic architecture of marine species. This study investigates the phylogeny and population genetics of two closely related syngnathid fishes, *Trachyrhamphus serratus* and *Trachyrhamphus longirostris*. We report the first record of *T. longirostris* along the mainland coast of China, with samples collected from Yantai, Kenting, Zhanjiang, and Beihai. Population genetic analyses reveal genetic differentiation within *T. longirostris*, which exhibits low levels of genetic diversity across all sampled populations compared to *T. serratus*. The star-like haplotype network and significantly negative neutrality test values collectively indicate a recent population expansion event in *T. longirostris*. This study offers important insights into the evolutionary dynamics and biogeographic patterning of syngnathid fishes, with clear implications for their conservation and management.

## Simple Summary

This study explores the genetic diversity, physical differences, and geographic distribution of two pipefish species in Chinese waters. We report the first finding of *Trachyrhamphus longirostris* along mainland China’s coast. The two species look different, mainly in their snout length. Their genetic patterns also contrast: *T. serratus* populations are genetically diverse and well-connected, while *T. longirostris* shows lower diversity and signs of a recent population spread. This research provides key information to help protect these pipefish in a changing environment.

## 1. Introduction

Pipefishes (Family Syngnathidae) are a distinctive group of nearshore, benthic fishes characterized by specialized morphological adaptations, including a toothless tubular mouth, male brood pouch incubation, and the absence of pelvic fins [1,2,3]. Their unique life-history traits, combined with limited reproductive output and restricted dispersal capabilities, render them particularly vulnerable to environmental change [4,5]. Consequently, syngnathids are increasingly recognized as flagship taxa for coastal marine conservation [6,7]. In the context of accelerating global environmental change and intensifying anthropogenic pressures, which are leading to widespread degradation of coastal habitats, understanding the status of these sensitive species is imperative [8,9,10]. The East Asian Seas region has the richest marine biodiversity in the world [11], hosts exceptional concentrations of critical habitats including coral reefs, mangroves, seagrass beds, and coastal wetlands. Notably, the coastal waters of this region harbor the highest species richness of syngnathids worldwide [12,13]. Despite their ecological significance and conservation value, comprehensive data on the population status and genetic resources of many syngnathid species, including those within this biodiversity hotspot, remain scarce [14]. A detailed assessment of species diversity and population structure is a fundamental prerequisite for developing effective conservation and management strategies [15].

The pipefish genus *Trachyrhamphus* currently comprises three morphologically similar species distributed along coastlines of the Indo-Pacific Ocean. While previous studies have investigated certain aspects of the phylogeny of *Trachyrhamphus* species [16,17,18], their geographical distribution and population status have received far less scientific attention. Available records remain sparse, and notably, the population trends of all three recognized *Trachyrhamphus* species are currently categorized as “Unknown” on the IUCN Red List (https://www.iucnredlist.org, accessed on 1 January 2026). Moreover, studies and documented observations of *Trachyrhamphus* species are limited, and distinguishing among the three species based on morphology alone is challenging. This combination of factors may lead to inaccuracies or biases in records made by scientists, fishers, and aquarium enthusiasts. Driven by intensifying climate change, marine biodiversity is currently subject to rapid, climate-driven transformations, occurring at a pace that exceeds those documented in terrestrial ecosystems [19]. In this context, understanding population connectivity, genetic structure, and demographic dynamics is crucial for assessing the climate vulnerability and future trajectories of marine species [20,21]. Such approaches have already been applied to other syngnathid fishes. For instance, a global assessment of syngnathid fishes indicates that a high number of threatened species are distributed in East and Southeast Asia, many of which likely face elevated extinction risks and therefore warrant urgent field research and comprehensive evaluation [5].

In this study, we document the first record of a *T. longirostris* population in the coastal waters of mainland China, thus reporting a new distribution for this species. We present morphological data for *T. longirostris* and its congener *T. serratus*. Furthermore, we amplified sequences from the mitochondrial cytochrome c oxidase subunit I gene regions of 131 *Trachyrhamphus* individuals from 9 geographic locations. Our objectives were to: (1) provide detailed morphological descriptions and diagnostic comparisons between the two co-distributed *Trachyrhamphus* species; (2) elucidate the phylogeographic relationship between *Trachyrhamphus* species and other syngnathid fishes; (3) assess the genetic diversity and population structure of the newly recorded *T. longirostris* population.

## 2. Materials and Methods

### 2.1. Sampling and DNA Extraction

**Figure 1:**
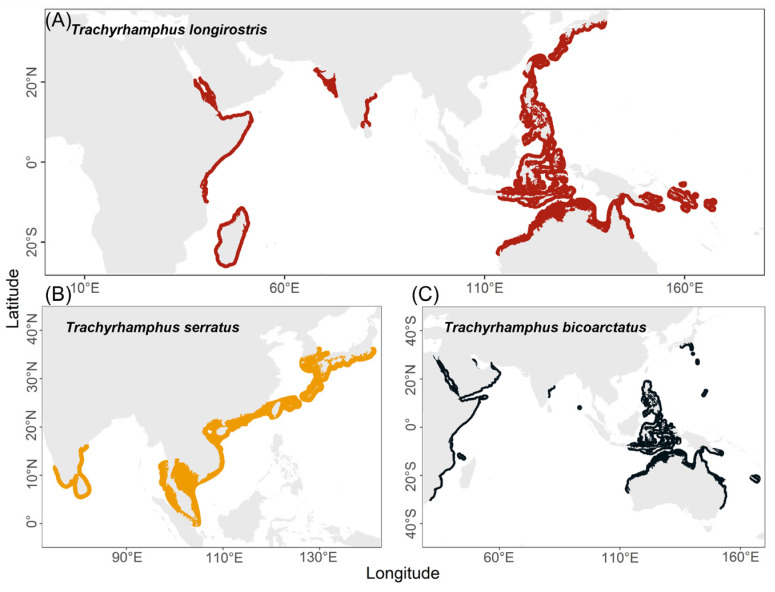
Geographical distributions of *Trachyrhamphus* species. (**A**) Geographical distribution of *T. longirostris* (shown in red); (**B**) Geographical distribution of *T. serratus* (shown in yellow); (**C**) Geographical distribution of *T. bicoarctatus* (shown in black).

Three *Trachyrhamphus* species have been described to date, comprising *T. serratus*, *T. longirostris*, and *T. bicoarctatus*. We gathered the geographical distributions (spatial ranges) data from the International Union for Conservation of Nature (IUCN) Red List of Threatened Species (Figure 1). A total of 131 *Trachyrhamphus* adult individuals were collected from the coastal waters of China, including 74 *T. serratus* and 57 *T. longirostris* (Table 1). All pipefish specimens were collected by researchers in collaboration with local fishermen. A small section of dorsal fin tissue was excised from each fresh specimen and preserved in 95% ethanol for subsequent DNA extraction. Concurrently, the following basic morphological measurements were recorded for each *Trachyrhamphus* individual: snout length (SnL), body height (BH), body width (BW), head length (HL), standard length (SL), and the ratios SnL/HL, BH/BW, and HL/SL. Genomic DNA was extracted from dorsal fin tissues used TIANamp Marine Animal DNA Kit (Tiangen, Dalian, China) according to the manufacturer’s instructions. The quality and integrity of the DNA samples were checked by agarose gel electrophoresis, and DNA concentrations were quantified using a Qubit 2.0 fluorometer (Thermo Fisher Scientific, Waltham, MA, USA).

### 2.2. Comparative Morphometric Analysis

In this study, principal component analysis was performed to examine morphological variation among the *Trachyrhamphus* specimens. The analysis was conducted using the following morphological variables: snout length (SnL), body height (BH), body width (BW), head length (HL), standard length (SL), and the ratios SnL/HL, BH/BW, and HL/SL. The resulting principal component scores were visualized using scatter plots to assess morphological differentiation between species and populations.

### 2.3. Primers, PCR, and Sequencing

A fragment of the mitochondrial cytochrome c oxidase subunit I (COI) gene was amplified using the specific primer pair: COIF 5′-TCGACTAATCATAAAGATATCGGCAC-3′ and reverse COIR 5′-TAGACTTCTGGGTGGCCAAAGAATCA-3′. PCR was performed in a T-100 thermocycler (Bio-Rad, Hercules, CA, USA) with TaKaRa LA Taq polymerase under the following program: initial denaturation at 94 °C for 3 min; 35 cycles of denaturation at 94 °C for 30 s, annealing at 55 °C for 30 s, and extension at 68 °C for 1 min; followed by a final extension at 68 °C for 10 min. PCR products were visualized on 1.5% agarose gels, purified using the E.Z.N.A. Gel Extraction Kit (Omega BioTek, Norcross, GA, USA), and sequenced bidirectionally on an ABI 3730 sequencer (Applied Biosystems, Foster City, CA, USA).

### 2.4. Phylogenetic and Population Genetic Analyses

Ultraconserved elements (UCEs) from 15 Syngnathidae species were obtained from a previously published study [13], and divergence time estimation was performed using MCMCtree [22]. Four calibration points were applied as constraints in the analysis: *Trachyrhamphus*–*Halicampus* (12.4–32.5 Mya), *Syngnathus*–*Halicampus* (27.4–55.3 Mya), and *Syngnathus schlegeli*–*Syngnathus scovelli* (10.2–23.5 Mya) from the TimeTree database [23], as well as the previously reported divergence between Nerophinae and Syngnathinae (49.9–67.1 Mya) [13].

All obtained COI sequences were aligned using MAFFT version 7 [24]. The best-fit nucleotide substitution model was selected with ModelTest 3.06 [25]. Maximum-likelihood (ML) phylogenetic analyses were conducted in IQ-TREE v3.1.1 [26]. The numbers of sequences (n), number of variable sites (ns), number of haplotypes (h), haplotype diversity (hd), nucleotide diversity (Pi), and average number of nucleotide differences (k) for each population were estimated using DnaSP version 5.10 program [27]. Haplotype median-joining network was constructed using the PopART software v1.7 [28].

Neutrality tests were performed using DnaSP version 5.10 program based on haplotype data derived from the mitochondrial cytochrome c oxidase subunit I (COI) gene sequences of *T. longirostris* and *T. serratus*. For each population, Fu’s *F_S_* and Tajima’s *D* statistics were calculated to assess deviations from neutral evolution under the assumption of constant population size. Significance levels were estimated via coalescent simulations (10,000 replicates). Negative values of both statistics suggest population expansion, while positive values may indicate population bottleneck. Pairwise mismatch distribution analysis was conducted in Arlequin v3.1 on combined data from all sampling locations to test for signals of past demographic expansion.

### 2.5. Statistical Analyses

Morphological measurements (Snout Length, Head Length, Body Height, Body Width, Standard Length, and their ratios) were analyzed using one-way analysis of variance (ANOVA) to test for significant differences among sampling locations. Prior to ANOVA, data normality and homogeneity of variances were assessed; when assumptions were met, post hoc multiple comparisons (Tukey’s HSD test) were performed to identify pairwise differences. Statistical significance was set at *p* < 0.05. Different superscript letters (a, b, c, d) assigned to values within the same column indicate statistically significant differences among locations; values sharing the same letter are not significantly different. Data are presented as mean ± standard deviation.

## 3. Results

### 3.1. Geographic Distribution and Phylogeny of Genus Trachyrhamphus

All three *Trachyrhamphus* species are widely distributed along coastlines of the Indo-Pacific Ocean (Figure 1). Among them, *T. longirostris* and *T. bicoarctatus* exhibit the broadest ranges, spanning from the western Indian Ocean to the central Pacific (Figure 1A,B). However, it is noticeable that there are no distribution records for *T. longirostris* in the Northwestern South China Sea and Sunda Shelf region, creating a gap in the middle of its geographical range. In contrast, the distribution of *T. serratus* is comparatively restricted, primarily concentrated in the western Pacific region. Its range includes the coast of mainland China, extending westward to the coastal waters of the Indian subcontinent (Figure 1C). Based on the overall distribution patterns, *T. longirostris* and *T. serratus* occupy distinct geographical ranges, revealing a clear and significant separation between the two species, particularly evident in the coastal waters of the western Pacific. *T. longirostris* is predominantly abundant in the Coral Triangle region, while *T. serratusis* distributed along the northern coast of the South China Sea, extending southward to the coastal waters of the Malay Peninsula.

**Figure 2:**
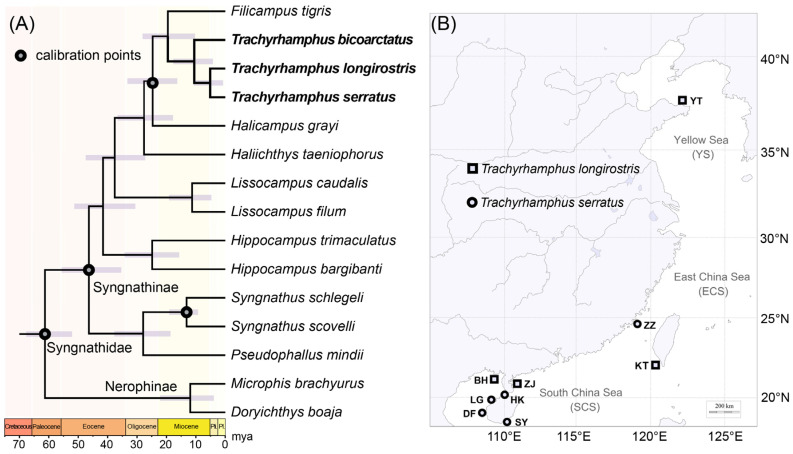
Phylogeny and collection site of *T. serratus* and *T. longirostris* on the coast of China. (**A**) Time-calibrated phylogenetic tree of 15 Syngnathidae species, constructed using ultraconserved elements (UCEs) obtained from a previously published study, with black circles indicating calibration points used in divergence time estimation. (**B**) Geographic distribution map showing collection localities of *T. serratus* and *T. longirostris*. YT (Yantai, 37.496048° N, 121.805047° E), KT (Kenting, 21.928298° N, 120.766592° E), BH (Beihai, 21.366139° N, 109.345198° E), ZJ (Zhanjiang, 21.173024° N, 110.725118° E), ZZ (Zhangzhou, 24.284292° N, 118.290845° E), HK (Haikou, 20.048229° N, 109.988700° E), LG (Lingao, 19.954869° N, 109.491338° E), DF (Dongfang, 19.092174° N, 108.561462° E), SY (Sanya, 18.185177° N, 109.761946° E).

The three *Trachyrhamphus* species (*T. longirostris*, *T. bicoarctatus*, and *T. serratus*) represent a monophyletic group within the family Syngnathidae (seahorses and pipefishes). The genus *Trachyrhamphus* diverged from its closest relatives during the Early Miocene period (Figure 2A). The split between the subfamilies Syngnathinae (which includes *Trachyrhamphus*) and Nerophinae occurred approximately 61 million years ago (Mya), placing this divergence shortly after the K-Pg boundary, a time of significant ecological recovery and evolutionary radiation after the mass extinction event. To further investigate the population genetic structure and genetic diversity of *Trachyrhamphus* species, we conducted field sampling across nine geographical locations in China’s coastal waters. Notably, we recorded the first occurrence of *T. longirostris* along the mainland coast of China, with population samples collected from Yantai, Kenting, Zhanjiang, and Beihai (Figure 2B).

### 3.2. Morphology Analysis of T. serratus and T. longirostris

**Figure 3:**
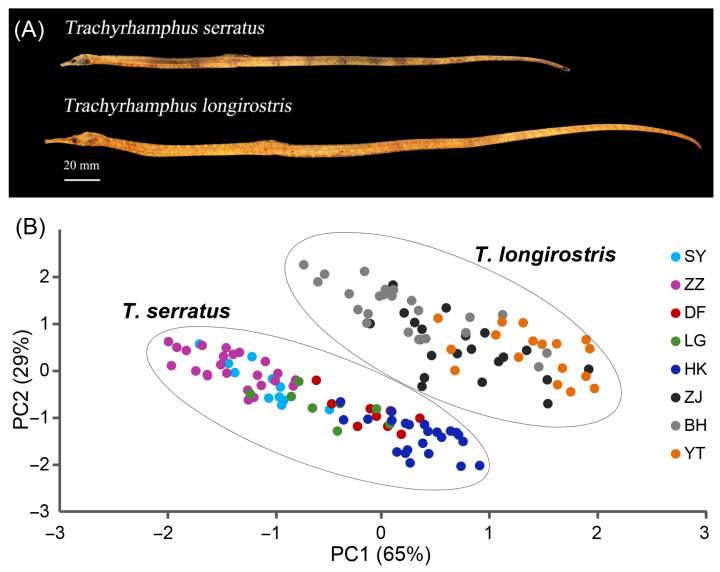
Morphology of *T. serratus* and *T. longirostris.* (**A**) Representative whole-body lateral images of adult specimens of *T. serratus* (top) and *T. longirostris* (bottom), illustrating key morphological differences; scale bar = 20 mm; (**B**) Principal component analysis (PCA) plot based on morphological measurements, displaying separation between *T. serratus* and *T. longirostris*.

We analyzed the morphological variables of *T. serratus* and *T. longirostris* (Figure 3A), including snout length (SnL), body height (BH), body width (BW), head length (HL), standard length (SL), and the ratios SnL/HL, BH/BW, and HL/SL (Table 1). The morphological data, together with the PCA results, provide clear evidence of the distinct physical characteristics that differentiate *T. serratus* and *T. longirostris*. The statistical analysis shows that *T. longirostris* exhibits significantly greater SnL and SL compared to *T. serratus*. Notably, *T. longirostris* possesses a relatively longer snout, as indicated by its higher SnL/HL ratio. PCA further reveals a clear and significant separation between the two species (Figure 3B). Moreover, morphological divergence is also observed among different populations within each species. In *T. longirostris*, for instance, the Yantai and Beihai populations show a certain degree of differentiation. Similarly, within *T. serratus*, the Zhangzhou and Haikou populations exhibit pronounced divergence, especially along PC1 (Figure 3B).

### 3.3. Genetic Diversity of Different Geographic Populations of T. serratus and T. longirostris

Genetic diversity indices, including haplotype diversity (hd), nucleotide diversity (Pi), and average number of nucleotide differences (k), are summarized in Table 2. The overall results indicate a disparity in genetic diversity between the two species, with all measured parameters consistently higher for *T. serratus* populations compared to those of *T. longirostris*. Populations of *T. serratus* exhibited relatively high levels of genetic diversity (Table 2). Haplotype diversity (hd) ranged from 0.857 ± 0.137 (LG) to 1.000 ± 0.063 (DF), The high number of haplotypes (h) and polymorphic sites (s) across all T. serratus populations further confirms the species’ overall high genetic variation.

In contrast, all populations of *T. longirostris* displayed lower genetic diversity. Haplotype diversity (hd) was moderate to low, ranging from 0.667 ± 0.163 (ZJ) to 0.857 ± 0.137 (BH). Nucleotide diversity (Pi) was consistently lower than in *T. serratus*, with values from 0.00225 ± 0.00065 (KT) to 0.00392 ± 0.00136 (ZJ). Correspondingly, the average number of nucleotide differences (k) was also lower. Although the Zhanjiang (ZJ) population exhibited the highest diversity indices within *T. longirostris* (Pi = 0.00392, k = 2.578), its values remained substantially below those observed in most *T. serratus* populations (Table 2).

### 3.4. Population Genetic Structure of T. serratus and T. longirostris

**Figure 4:**
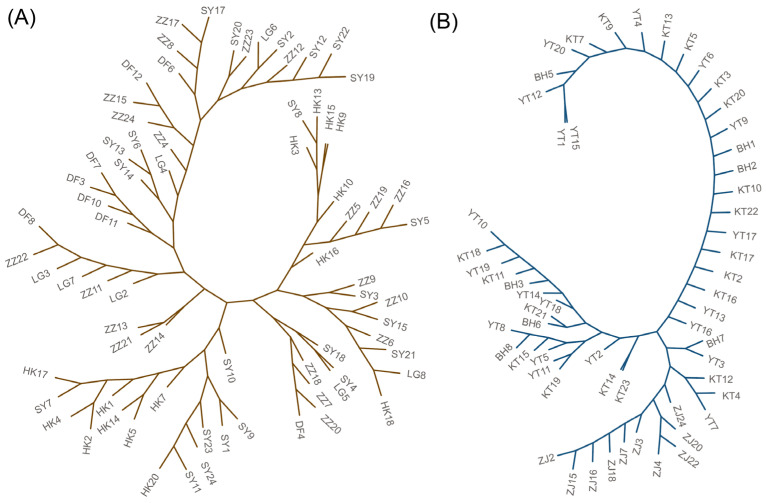
Maximum-likelihood phylogenetic tree of populations in *T. serratus* and *T. longirostris* based on the COI gene. (**A**) ML phylogenetic tree of *T. serratus* (n = 74) populations; (**B**) ML phylogenetic tree of *T. longirostris* (n = 57) populations.

Phylogenetic analysis based on the mitochondrial COI gene reveals distinct evolutionary patterns between the two species (Figure 4A,B). Within each species, the maximum likelihood tree indicates minimal genetic differentiation among geographically separated populations. For *T. serratus* (Figure 4A), individuals from various locations are broadly intermixed within the clade, with no clear branches corresponding to specific geographic regions. This pattern suggests high gene flow or recent population expansion with insufficient time for lineage sorting. A more pronounced pattern is observed in *T. longirostris* (Figure 4B). The overall topology is characterized by short internal branches; however, individuals from the Zhanjiang (ZJ) region form a distinct, moderately supported sub-clade that separate from the main cluster containing all other *T. longirostris* specimens (e.g., from YT, KT, BH). Furthermore, the phylogenetic placement of the newly recorded Chinese *T. longirostris* samples within the broader *T. longirostris* clade confirms its taxonomic identity (Figure 4A), which may suggests a recent expansion or migration event from its core Indo-Pacific range.

### 3.5. Haplotype Networks of T. serratus and T. longirostris

**Figure 5:**
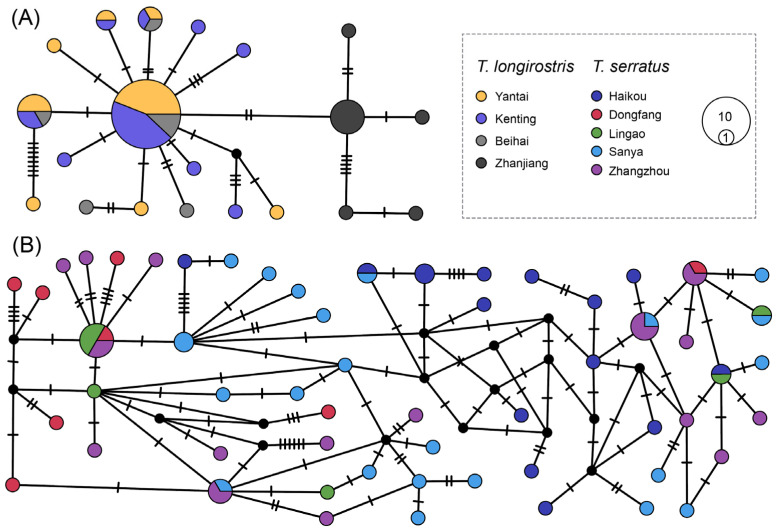
Haplotype median-joining network based on COI gene for *T. longirostris* (**A**) and *T. serratus* (**B**) with pie charts showing haplotype composition by geographic origin. The size of the circles represents the number of individuals within each haplotype and short lines indicate the number of mutational steps between haplotypes.

The median-joining haplotype network provides a finer-scale view of intraspecific genetic relationships of *T. serratus* and *T. longirostris*. The two species exhibit markedly different network topologies, reflecting contrasting population histories and genetic structures. In *T. longirostris* (Figure 5A), the network is characterized by a star-like structure, with a central, high-frequency haplotype shared among populations from Yantai, Kenting, and Beihai. Several low-frequency haplotypes, differing by one to a few mutational steps, radiate from this central node. In contrast, the haplotype representing the Zhanjiang population, depicted as a solid black circle, is distinctly separate. This structural isolation of the Zhanjiang haplotype revealed a notable genetic distinction from other *T. longirostris* populations, and this observation is fully congruent with the phylogenetic results (Figure 4B), where the Zhanjiang samples formed a unique clade, thereby confirming that the Zhanjiang population represents a relatively independent genetic lineage within the species.

In contrast, the network for *T. serratus* displays a more complex and reticulate pattern. Multiple haplotypes of similar frequency, represented by similarly sized circles corresponding to different localities, are interconnected in a web-like structure (Figure 5B). This topology suggests a longer demographic history, higher levels of ancestral polymorphism, and/or greater gene flow among populations, contrasting sharply with the signature of recent expansion or founder effect seen in *T. longirostris*.

### 3.6. Neutrality Tests and Historical Demography of T. serratus and T. longirostris

To further elucidate the population evolutionary history of *T. longirostris* and *T. serratus*, neutrality tests (Fu’s *F_S_* and Tajima’s *D*) were conducted based on haplotype data. Among the four populations of *T. longirostris* (Yantai, Kenting, Beihai, Zhanjiang), most exhibited significant negative values of both Tajima’s *D* and Fu’s *F_S_* (Table 3), indicating signatures of recent population expansion. Notably, the Zhanjiang population of *T. longirostris* stands out with non-significant neutrality tests, aligning with its unique genetic position in both haplotype networks and phylogenetic trees—likely reflecting a historically stable or isolated demographic trajectory. The results for the five populations of *T. serratus* (Zhanjiang, Haikou, Dongfang, Lingao, Sanya) revealed that the Tajima’s *D* values for all populations were not statistically significant (*p* > 0.05) (Table 3). This pattern parallels the haplotype network results, in which *T. serratus* exhibits a complex, reticulate haplotype structure, in contrast to the star-like network typical of recent demographic expansion.

**Figure 6:**
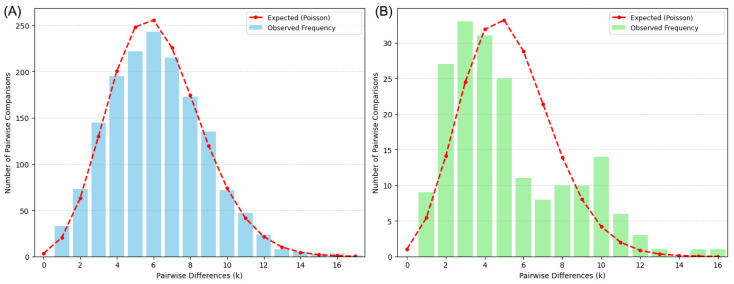
Mismatch distribution analysis of historical demography for *T. serratus* (**A**) and *T. longirostris* (**B**).

Demographic history changes were analyzed for *T. serratus* and *T. longirostris* populations using mismatch distributions. The expected distribution exhibits a smooth unimodal curve, which aligns with the inference drawn from the neutrality test. The haplotype mismatch distribution curves for the two pipefish species display a unimodal Poisson pattern (Figure 6), consistent with the expected distribution under the population expansion model. This suggests that their populations may have undergone expansion.

## 4. Discussion

Syngnathid fishes have long been a focus of conservation biology, evolutionary biology, and biogeography due to their distinctive biological traits and complex evolutionary histories [29,30]. Characterized by limited dispersal ability, high habitat specificity, and unique reproductive strategies (e.g., male pregnancy), they are particularly sensitive to environmental changes and often exhibit pronounced genetic structuring among geographically isolated populations [9,31,32]. In this study, all three *Trachyrhamphus* species are widely distributed along the coastlines of the Indo-Pacific Ocean. This differs from other members of the Syngnathidae family, such as seahorses and certain other pipefish species (e.g., common seadragons), which tend to exhibit more restricted or localized distributions [9,32]. The distinct geographical distributions of *T. longirostris* and *T. serratus* in the western Pacific region provide important insights into their evolutionary histories and ecological adaptations. *T. longirostris* exhibits a broad Indo-Pacific distribution, with its core abundance centered in the Coral Triangle region, while *T. serratus* distributed primarily along the coast of the South China Sea. Notably, *T*. *longirostris* shows a discontinuous distribution pattern, as no records exist across the middle of its geographic range. Such disjunct distributions are indeed common in marine fishes [33]. This biogeographic pattern is widespread across the Tree of Life, though the underlying mechanisms remain debated. It is typically attributed to two competing hypotheses: dispersal and vicariance. The discovery of *T. longirostris* as a new record along the mainland coast of China has important conservation implications. The species’ relatively low genetic diversity in this region suggests that these populations may be more vulnerable to environmental changes, habitat loss, or other anthropogenic pressures [34]. The Zhanjiang population represents a unique evolutionary lineage that has undergone relative isolation, accumulating distinct mutations not found in other conspecifics. It thus constitutes an important reservoir of genetic diversity for the species, warranting prioritized conservation attention. Future studies should focus on quantifying the extent of its genetic distinctiveness and assessing its specific conservation priority to inform management strategies. The loss of this population would result in the permanent loss of unique evolutionary potential. Concerns regarding the conservation of unique genetic lineages have similarly been documented in studies of other syngnathid fish species. Stiller et al. (2023) examined the population genetic structure of the *Phyllopteryx taeniolatus*, identified a phylogeographic break across a former land bridge within its range, and highlighted conservation concerns for this species [9].

The contrasting patterns of genetic diversity between *T. serratus* and *T. longirostris* reveal distinct demographic histories. *T. serratus* populations exhibit relatively high levels of genetic diversity, and the complex, reticulate haplotype network structure further supports a longer demographic history with stable population sizes and/or extensive gene flow among populations. In contrast, *T. longirostris* populations show lower genetic diversity across all measured parameters. The star-like haplotype network, characterized by a central high-frequency haplotype with radiating low-frequency haplotypes, is a classic signature of a recent population expansion following a bottleneck or founder event [35,36]. This pattern suggests that *T. longirostris* may have experienced a recent demographic expansion into the western Pacific region. Ocean currents, especially the Kuroshio Current and its branches, likely played an important role in facilitating the species’ dispersal, potentially enabling the northward transport of larvae or adults toward coastal China from regions such as the Coral Triangle—a biodiversity hotspot regarded as a potential source area for numerous Indo-Pacific marine taxa [37,38]. While the present study is confined to samples from Chinese coasts and public databases, clarifying the species’ exact origin and dispersal pathways will necessitate broader phylogeographic and population genetic analyses in the future. In another study, Bertola et al. (2020) assessed the genetic structure of five sympatric syngnathid species in the Gulf of Mexico, confirming the contribution of ocean currents to population structure [39]. The low genetic diversity observed in the Chinese coastal populations is consistent with this scenario, as newly colonized populations often exhibit reduced genetic variation due to founder effects and genetic drift [40,41]. This study provides a foundation for several future research directions. First, the use of nuclear markers, such as single nucleotide polymorphisms (SNPs) derived from RAD-seq or whole-genome resequencing, would provide additional insights into the population structure and demographic history of both species, complementing the mitochondrial DNA data presented here. Second, ecological studies investigating habitat preferences, feeding ecology, and reproductive biology would help understand the factors driving the observed distribution patterns and genetic structure. Finally, ongoing monitoring of population trends and genetic diversity is essential to assess the long-term viability of these species in the face of ongoing environmental changes.

## 5. Conclusions

This study provides comprehensive insights into the phylogeography, morphological variation, and genetic structure of *Trachyrhamphus* species in Chinese coastal waters. The discovery of *T. longirostris* as a new record along mainland China represents a significant biogeographic finding. Morphological analyses demonstrate clear differentiation between the two species, particularly in snout length and standard length, with *T. longirostris* possessing a relatively longer snout. Population genetic structure analyses reveal contrasting patterns: *T. serratus* populations exhibit high genetic diversity with complex haplotype networks, suggesting stable demographic histories and extensive gene flow, whereas *T. longirostris* populations show lower diversity with star-like haplotype networks, indicating recent population expansion. These findings provide critical baseline data on the population distribution of these pipefish species, thus informing effective conservation strategies in the face of ongoing environmental changes.

## Figures and Tables

**Table 1:** Morphological data analysis of different *Trachyrhamphus* populations.

Location/Morphological Characteristics	SnL	HL	BH	BW	SL	SnL/HL	BH/BW	HL/SL
SY	6.42 ± 0.29 ^a^	10.82 ± 0.42 ^ab^	5.97 ± 0.35 ^ab^	5.10 ± 0.46 ^ab^	98.23 ± 3.57 ^a^	0.59 ± 0.04 ^a^	1.18 ± 0.10 ^a^	0.11 ± 0.01 ^ab^
ZZ	6.39 ± 0.29 ^a^	10.58 ± 0.37 ^a^	5.47 ± 0.40 ^a^	4.90 ± 0.46 ^a^	97.88 ± 3.96 ^a^	0.60 ± 0.03 ^a^	1.12 ± 0.09 ^a^	0.11 ± 0.01 ^a^
DF	7.17 ± 0.28 ^a^	11.95 ± 0.38 ^c^	6.92 ± 0.44 ^cd^	5.86 ± 0.37 ^cd^	98.70 ± 2.49 ^a^	0.60 ± 0.03 ^a^	1.18 ± 0.08 ^a^	0.12 ± 0.01 ^cd^
LG	6.50 ± 0.56 ^a^	11.32 ± 0.71 ^b^	6.52 ± 0.56 ^bc^	5.81 ± 0.28 ^cd^	97.93 ± 3.91 ^a^	0.57 ± 0.04 ^a^	1.12 ± 0.09 ^a^	0.12 ± 0.01 ^abc^
HK	7.06 ± 0.63 ^a^	12.67 ± 0.49 ^d^	7.19 ± 0.49 ^d^	6.21 ± 0.35 ^d^	97.76 ± 4.35 ^a^	0.56 ± 0.04 ^a^	1.16 ± 0.08 ^a^	0.13 ± 0.01 ^d^
ZJ	12.36 ± 1.08 ^b^	12.80 ± 0.63 ^d^	6.45 ± 0.81 ^bc^	5.51 ± 0.61 ^bc^	107.34 ± 5.93 ^b^	0.97 ± 0.08 ^bc^	1.17 ± 0.12 ^a^	0.12 ± 0.01 ^bc^
BH	12.34 ± 0.69 ^b^	11.98 ± 0.71 ^c^	5.79 ± 0.58 ^a^	5.09 ± 0.61 ^ab^	111.93 ± 6.52 ^bc^	1.03 ± 0.05 ^c^	1.14 ± 0.05 ^a^	0.11 ± 0.01 ^a^
YT	12.76 ± 1.28 ^b^	13.21 ± 0.44 ^d^	7.08 ± 0.60 ^cd^	6.38 ± 0.55 ^d^	113.32 ± 6.37 ^c^	0.96 ± 0.09 ^b^	1.11 ± 0.10 ^a^	0.12 ± 0.01 ^bc^

SnL: Snout Length; HL: Head Length; BH: Body Height; BW: Body Width SL: Standard Length; Data are presented as mean ± standard deviation. Different superscript letters (a, b, c, d) within the same column indicate statistically significant differences among locations based on one-way ANOVA followed by post hoc multiple comparisons (*p* < 0.05). Values sharing the same letter are not significantly different.

**Table 2:** Genetic diversity of different *Trachryhamphus* populations.

Species	Location	n	s	h	hd	Pi	k
*T. serratus*	HK	15	22	14	0.990 ± 0.028	0.00790 ± 0.00104	5.267
	DF	8	16	8	1.000 ± 0.063	0.00792 ± 0.00116	5.286
	LG	7	7	5	0.857 ± 0.137	0.00387 ± 0.00101	2.571
	SY	23	32	19	0.984 ± 0.017	0.00741 ± 0.00091	4.893
	ZZ	21	30	18	0.964 ± 0.025	0.00780 ± 0.00124	5.192
*T. longirostris*	BH	7	7	5	0.857 ± 0.137	0.00341 ± 0.0094	2.238
	KT	20	13	9	0.705 ± 0.111	0.00225 ± 0.0065	1.479
	YT	20	15	8	0.695 ± 0.108	0.00279 ± 0.00116	1.831
	ZJ	10	9	5	0.667 ± 0.163	0.00392 ± 0.00136	2.578

n represents the number of sequences, s indicates the number of variable sites, h indicates the number of haplotypes, hd stands for haplotype diversity (haplotype diversity ± standard deviation), Pi represents nucleotide diversity (nucleotide diversity ± standard deviation), and k indicates the average number of nucleotide differences.

**Table 3:** Summary of neutrality tests (Fu’s *F_S_* and Tajima’s *D*) for *T. longirostris* and *T. serratus* populations.

Species	Population	Tajima’s *D*	Fu’s *F_S_*
*T. longirostris*	YT	−1.689 *	−4.496 *
	KT	−1.723 *	−6.839 **
	BH	−1.358 ***	−2.175 ^ns^
	ZJ	0.461 ^ns^	−1.481 ^ns^
*T. serratus*	ZZ	−1.146 ^ns^	−13.079 ***
	HK	−1.158 ^ns^	−10.057 ***
	DF	−1.000 ^ns^	−3.752 *
	LG	0.083 ^ns^	−2.004 ^ns^
	SY	−1.331 ^ns^	−21.298 ***

Significance levels: *: 0.01 < *p* < 0.05, **: 0.001 < *p* < 0.01, ***: *p* < 0.001, ^ns^: *p* > 0.05.

## Data Availability

Data are contained within the article; further inquiries can be directed to the corresponding author.
